# A case of kidney graft injury during cesarean section in a pancreas and kidney transplantation recipient

**DOI:** 10.20407/fmj.2024-008

**Published:** 2024-10-31

**Authors:** Noriko Aida, Eiji Nishio, Takao Sekiya, Naohiro Aida, Taihei Ito, Takashi Kenmochi, Haruki Nishizawa

**Affiliations:** 1 Women’s Health Clinic Kariyaginza, Kariya, Aichi, Japan; 2 Department of Obstetrics and Gynecology, Fujita Health University, School of Medicine, Toyoake, Aichi, Japan; 3 Department of Transplantation and Regenerative Medicine, Fujita Health University, School of Medicine, Toyoake, Aichi, Japan

**Keywords:** Kidney and pancreas transplantation, Kidney graft injury, Delivery, Uterine fundal pressure maneuvers

## Abstract

Although organ transplantation is becoming general practice, little is known about the safety of delivery. This is the first known case that describes injury to the kidney by the uterine fundal pressure maneuver during cesarean section in a pancreas and kidney transplant recipient. A 40-year-old pregnant woman (gravida 0, para 0) was referred to our clinic. She had undergone living donor kidney transplantation 11 years earlier and brain-dead donor pancreas transplantation 1 year earlier owing to type 1 diabetes. Cesarean section was indicated when the patient’s blood pressure was 150/100 mmHg at 37 weeks. We pushed the uterine fundus during delivery of the infant, with our usual caution. Serum creatinine levels were 1.6–2.6 mg/dl postoperatively. As this elevation was considered to be due to kidney graft dysfunction, we performed computed tomography, which revealed a hematoma around the kidney graft. Fifteen days after the cesarean section, surgical removal of the hematoma was performed by the transplant surgery team. Following hematoma removal, the serum creatinine level decreased to <1.4 mg/dl. We present a case of kidney graft injury during cesarean section in a pancreas and kidney transplant recipient.

## Introduction

Pancreas and kidney transplantation is an established treatment for end-stage renal disease due to type 1 diabetes. Pancreas and kidney transplantations comprise 83% simultaneous kidney and pancreas transplantation, 12% pancreas after renal transplantation, and 5% pancreatic transplantation alone.^1^ A recent meta-analysis reported that pregnancies following kidney transplantation are associated with increased risks of adverse events, such as preeclampsia, cesarean section, gestational diabetes, and preterm delivery.^2–5^ Although organ transplantation is becoming general practice, little is known about the safety of delivery after transplantation. This is the first known case that describes injury to the kidney by the uterine fundal pressure maneuver during cesarean section in a pancreas and kidney transplant recipient.

## Case report

**Figure 1:**
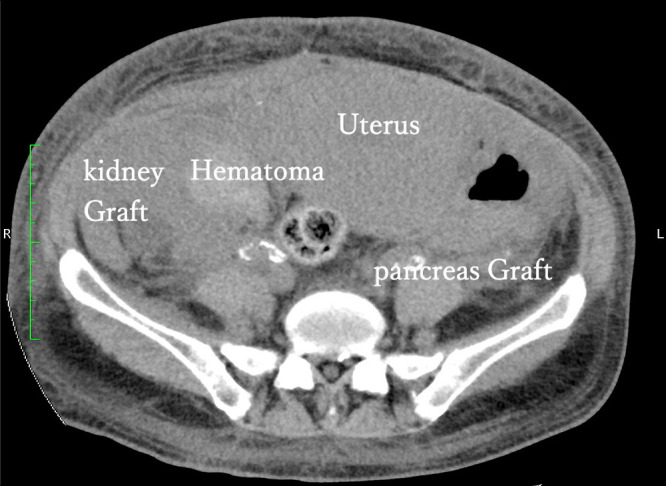
Computed tomography (transverse section) showing a hematoma around the kidney graft. The pancreas graft was intact.

**Figure 2:**
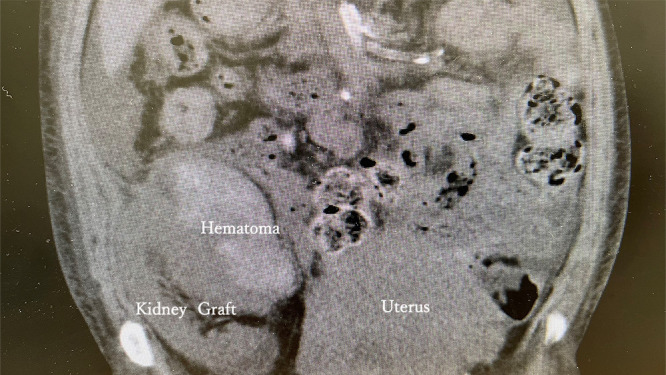
Computed tomography (vertical section) showing a hematoma around the kidney graft.

The patient provided written informed consent for the publication of her data, and the data have been fully anonymized to avoid patient identification. A 40-year-old pregnant woman (gravida 0, para 0) was referred to our clinic. She had undergone living donor kidney transplantation 11 years earlier and brain-dead donor pancreas transplantation 1 year earlier due to type 1 diabetes. The baseline serum creatinine levels were 0.9–1.5 mg/dl from the initial stage to 30 weeks’ gestation. At 31 weeks, the serum creatinine level increased to 1.5–2 mg/dl and blood pressure increased to 160/100 mmHg, indicating gestational hypertension. Therefore, cesarean section was planned after consultation with the patient and the transplant surgery team. Cesarean section was indicated when the patient’s blood pressure was 150/100 mmHg at 37 weeks’ gestation. A vertical incision was made and extended to the pubic symphysis. The fascia was then incised vertically. The surgical incision into the uterus was performed as a transverse incision. We pushed the fundus of the uterus to deliver the infant, with our usual caution. A viable infant with an Apgar score of 9 weighing 2140 g was delivered. No blood product transfusion was necessary. One day after cesarean section, the patient’s systolic blood pressure increased to >180 mmHg and the diastolic blood pressure increased to >110 mmHg. Additionally, blood hemoglobin had decreased from 9.1 g/dl to 6.9 g/dl, and serum creatinine levels were 1.6–2.6 mg/dl. The elevated serum creatinine levels indicated hypovolemia with prerenal failure. As this condition was considered to be due to kidney graft dysfunction, we performed computed tomography, which revealed a hematoma around the kidney graft and an intact pancreas graft (Figure 1 and 2). Fifteen days after the cesarean section, surgical removal of the hematoma was performed by the transplant surgery team. Postoperatively, the serum creatinine level decreased to <1.4 mg/dl, and the patient was discharged from the hospital.

## Discussion

There is no true consensus regarding the method of delivery for patients who have undergone kidney transplantation. In these patients, the cesarean section rate is higher (62.6%, 95% confidence interval: 57.6–67.3)^6^ compared with a rate of 32.8% in the general population in the USA.^7^ Cesarean section is not always safe in kidney transplant recipients. Gordon and Tatsis reported shearing-force injury to a kidney graft during repeat cesarean section, due to adherence of the organ to the overlying fascia.^8^

The uterine fundal pressure maneuver is widely performed during the second stage of labor to expedite delivery or to increase the expulsive force of the uterus in situations requiring urgent delivery.^9^ However, performing this maneuver during the second stage of labor increases the risk of severe perineal laceration.^10^ For this reason, the uterine fundal pressure maneuver has the potential to induce injury to the transplanted kidney. In our patient, the anatomic location of the kidney graft was lower and closer to the uterus compared with the anatomic location of her native kidney. Therefore, a hematoma developed due to pressure on the kidney graft. The cause of the hematoma was considered to be mechanical damage sustained during the uterine fundal pressure maneuver; however, high blood pressure can also cause kidney damage.

In a search of PubMed, we identified no reports of renal injuries with vaginal and cesarean deliveries among normal patients, with the uterine fundal pressure maneuver. In our experience, pushing on the uterine fundus should be performed carefully, whether during vaginal delivery or cesarean section. To avoid injury to a transplanted kidney, we should determine the associated anatomic changes in the location of the kidneys. Identifying the location of the transplanted kidney with magnetic resonance imaging and ultrasonography is important to prevent injury before cesarean section.

In conclusion, we presented a case of kidney graft injury during cesarean section in a pancreas and kidney transplant recipient. The uterine fundal pressure maneuver must be performed carefully in all situations.

## Data Availability

The authors confirm that the data supporting the findings of this study are available within the article and/or its supplementary materials.
